# Comparative Analysis of the Cutaneous Microbiome in Psoriasis Patients and Healthy Individuals—Insights into Microbial Dysbiosis: Final Results

**DOI:** 10.3390/ijms251910583

**Published:** 2024-10-01

**Authors:** Diana Sabina Radaschin, Alina Viorica Iancu, Alexandra Mariana Ionescu, Gabriela Gurau, Elena Niculet, Florin Ciprian Bujoreanu, Cristina Beiu, Alin Laurentiu Tatu, Liliana Gabriela Popa

**Affiliations:** 1 Department of Dermatology, “Saint Parascheva” Infectious Disease Clinical Hospital, 800179 Galati, Romania; diana.radaschin@ugal.ro (D.S.R.); florin.bujoreanu@ugal.ro (F.C.B.); 2 Department of Clinical Medical, Faculty of Medicine and Pharmacy, “Dunarea de Jos” University, 800008 Galati, Romania; 3 Multidisciplinary Integrated Centre of Dermatological Interface Research Centre (MICDIR), “Dunarea de Jos” University, 800008 Galati, Romania; 4 Department of Morphological and Functional Sciences, Faculty of Medicine and Pharmacy, “Dunarea de Jos” University, 800008 Galati, Romania; alina.iancu@ugal.ro (A.V.I.); gabriela.gurau@ugal.ro (G.G.); elena.niculet@ugal.ro (E.N.); 5 Faculty of Sciences and Environment, “Dunarea de Jos” University, 800008 Galati, Romania; alexaione96@yahoo.com; 6 Dermatology Department, Carol Davila University of Medicine and Pharmacy, 030167 Bucharest, Romania; liliana.popa@umfcd.ro

**Keywords:** psoriasis, skin microbiome, dysbiosis, inflammatory skin diseases

## Abstract

Psoriasis is one of the most frequent chronic inflammatory skin diseases and exerts a significant psychological impact, causing stigmatization, low self-esteem and depression. The pathogenesis of psoriasis is remarkably complex, involving genetic, immune and environmental factors, some of which are still incompletely explored. The cutaneous microbiome has become more and more important in the pathogenesis of inflammatory skin diseases such as acne, rosacea, atopic dermatitis and psoriasis. Dysbiosis of the skin microbiome could be linked to acute flare ups in psoriatic disease, as recent studies suggest. Given this hypothesis, we conducted a study in which we evaluated the cutaneous microbiome of psoriasis patients and healthy individuals. In our study, we collected multiple samples using swab sampling, adhesive tape and punch biopsies. Our results are similar to other studies in which the qualitative and quantitative changes found in the cutaneous microbiome of psoriasis patients are different than healthy individuals. Larger, standardized studies are needed in order to elucidate the microbiome changes in psoriasis patients, clarify their role in the pathogenesis of psoriasis, decipher the interactions between the commensal microorganisms of the same and different niches and between microbiomes and the host and identify new therapeutic strategies.

## 1. Introduction

Psoriasis is one of the most frequent chronic inflammatory skin diseases, its prevalence ranging from 0.5 to 11.4%, increasing with distance from the Equator [1]. Far from being solely a cosmetic issue, psoriasis exerts a significant psychological impact, causing stigmatization, low self-esteem and depression [2]. Psoriasis affecting special areas, such as the palms and soles, is often associated with functional impotence, while widespread psoriasis may lead to local and systemic complications. A series of comorbidities such as psoriatic arthritis, inflammatory bowel disease, ocular manifestations, diabetes, hypertension, and dyslipidemia are common in psoriasis patients [3]. The bidirectional relationship between psoriasis and obesity has also been thoroughly analyzed [4]. In addition, psoriasis represents an independent risk factor for cardiovascular disease, being associated with premature and accelerated atherosclerosis and with an increased risk of acute cardiovascular and cerebrovascular events [3]. With the introduction of highly effective biologic and small-molecule therapies, clinicians’ and patients’ treatment expectations have greatly risen, as complete clinical remission is achieved in the majority of psoriasis patients. Nevertheless, most patients eventually develop resistance to such therapeutic agents, requiring novel treatments. In addition, no therapy is curative, with specific treatment generally being required for indefinite periods of time, leading to potential adverse effects, especially those associated with long-term immunosuppression and considerable economic costs. The pathogenesis of psoriasis is remarkably complex, involving genetic, immune and environmental factors, some of which are still incompletely explored. Therefore, psoriasis continues to represent an appealing research topic in a continuous effort to identify modifiable risk factors for this disease and new therapeutic targets. As the importance of the intestinal microbiome became obvious in numerous chronic inflammatory diseases [5], the attention of researchers turned toward the influence of gut and skin microbiomes on the development of psoriasis, its exacerbations and response to treatment.

According to the definition proposed by Joshua Lederberg in 2001, the cutaneous microbiome represents the totality of cutaneous microorganisms that are harbored by the human skin [6]. The skin microbiome is composed of commensal microorganisms that reside on the human skin, transient microorganisms that are temporarily found on the skin and pathogenic agents that could influence the cutaneous homeostasis [7]. Bacteria, fungi, viruses and archae are part of the human microbiome. The major phyla found on human skin are Actinobacteria, Proteobacteria, Firmicutes and Bacteroidetes [8].

The relationship between the skin microbiome and psoriasis has become a subject of great interest, given the implications of different pathogens in the course of psoriasis. In genetically predisposed individuals, upper respiratory tract infections with group A beta-hemolytic streptococci act as triggers for guttate psoriasis in younger populations. Given the molecular mimicry between M proteins of Streptococcus spp. and keratin 1, the developing of an acute flare of guttate psoriasis, especially in children, is possible due to the inflammatory response generated by an increased population of Th1 lymphocytes [9].

TLR-9 on plasmacytoid dendritic cells (pDCs) binds complexes formed by LL-37 cathelicidins and the DNA of apoptotic keratinocytes, causing pDCs to secrete significant amounts of IFN-α, which in turn induces myeloid dendritic cells (mDCs) to mature and become activated [10]. Furthermore, by activating pDCs by TLR-7 and mDCs through TLR-8, LL-37—RNA complexes cause the release of TNF-α, IL-12, IL-23, and inducible nitric oxide synthase (iNOS). The main mediators of the pathogenic inflammatory cascade of psoriasis, Th1 and Th 17 cells, are produced when activated mDCs migrate to the local lymph nodes and induce differentiation of naive T cells [11].

More and more compelling evidence points to the role skin and gut dysbiosis and loss of immune tolerance to cutaneous and intestinal microorganisms play in the pathogenesis of psoriasis [12,13,14,15]. It is widely acknowledged that alterations of the skin and gut microbiomes generate a proinflammatory state, increased oxidative stress and altered DNA repair, but the precise mechanisms underlying the implication of dysbiosis in psoriasis pathogenesis are still to be uncovered [16,17]. Hence, modulation of the skin and gut microbiomes holds particular theoretical promise and is currently under investigation.

We wish to present the results of our study that aimed to characterize the skin microbiome in psoriasis patients compared with healthy controls in an attempt to identify microbial biomarkers related to the development, exacerbations, severity of the skin disease and response to treatment.

## 2. Results

**Figure 1:**
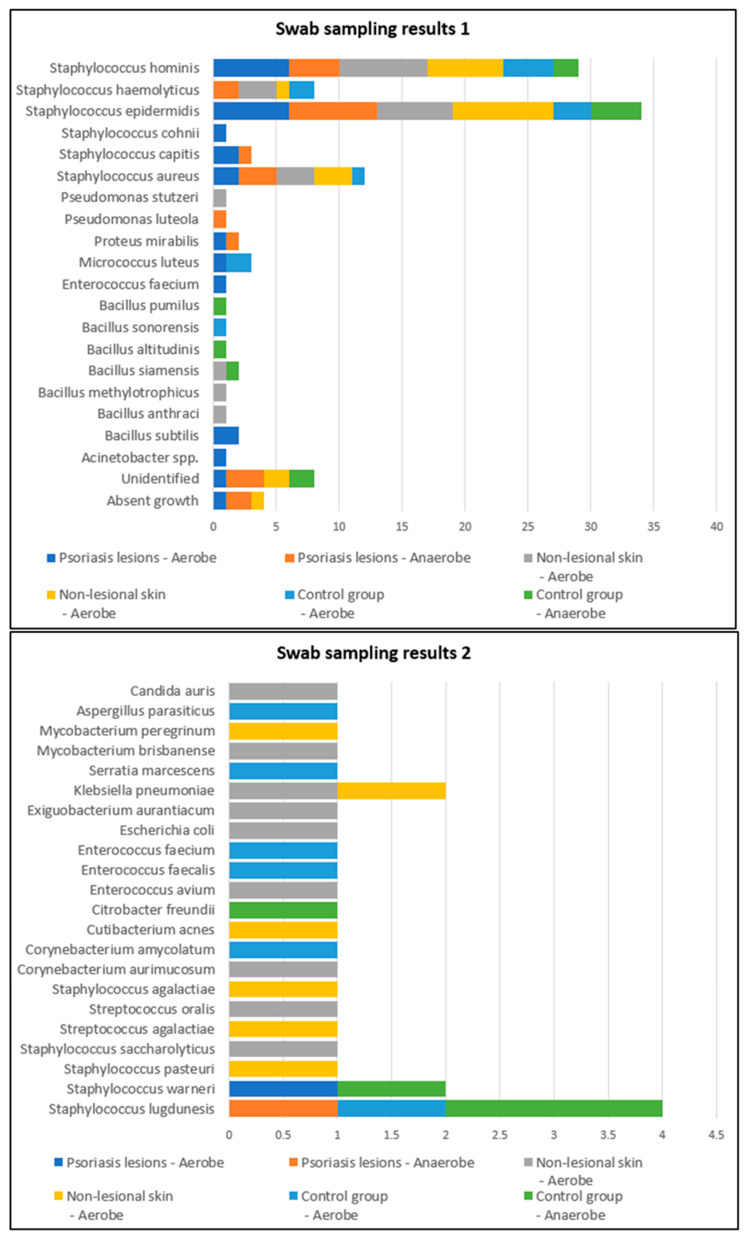
Swab sampling results from lesional skin, nonlesional sites in psoriasis patients and control group—bacterial species isolated in aerobe and anaerobe.

Swab samples were collected from nine different body regions from psoriatic plaques, mostly elbows and anterior trunk. Swab samples collected from nonlesional sites were mostly from the axillary and inguinal folds. In the active group, swab samples collected from lesional and perilesional skin and inoculated in aerobe and anaerobe mediums yielded the most positive culture results for *Staphylococcus epidermidis* and *Staphylococcus hominis* (Figure 1). Regarding the control group, swab sampling revealed less bacterial growth than the psoriatic group. Swab samples were prelevated from the axillary and inguinal folds (Figure 1).

**Figure 2:**
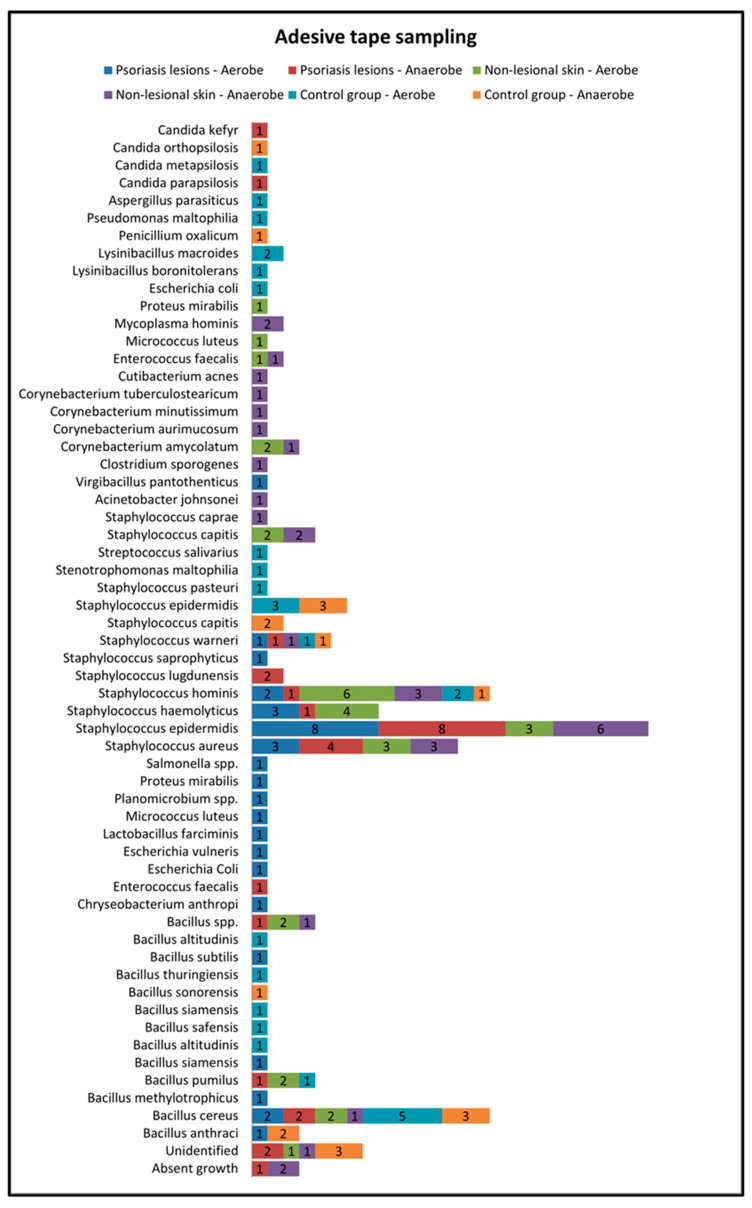
Adhesive tape sampling results from lesional skin, nonlesional sites in psoriasis patients and control group—bacterial species isolated in aerobe and anaerobe.

Skin sampling using adhesive tape was performed mostly from psoriatic lesions located on the elbows and knees and resulted in the most abundant bacterial growth. In the active group, cultures of the samples collected from lesional areas revealed 32 bacterial strains (20 in the aerobe culture medium and 12 in the anaerobe culture medium), the most frequent being *Staphylococcus epidermidis* and *Staphylococcus aureus* (Table 1). Adhesive-tape-stripping samples from the nonlesional skin of psoriasis patients and from the control group were mostly collected from the posterior trunk, calf and inguinal folds (Figure 2).

The samples collected through adhesive tape stripping from the nonlesional skin of psoriatic patients revealed a higher diversity of bacteria species compared with samples taken from psoriasis lesions and the control group. The most frequently isolated bacteria were *Staphylococcus hominis*, *Staphylococcus haemolyticus*, *Staphylococcus aureus* and *Staphylococcus epidermidis*.

On the other hand, in the control group, the most frequently isolated bacteria from samples collected using adhesive tape were *Bacillus cereus* and *Staphylococcus epidermidis* (Figure 2).

**Figure 3:**
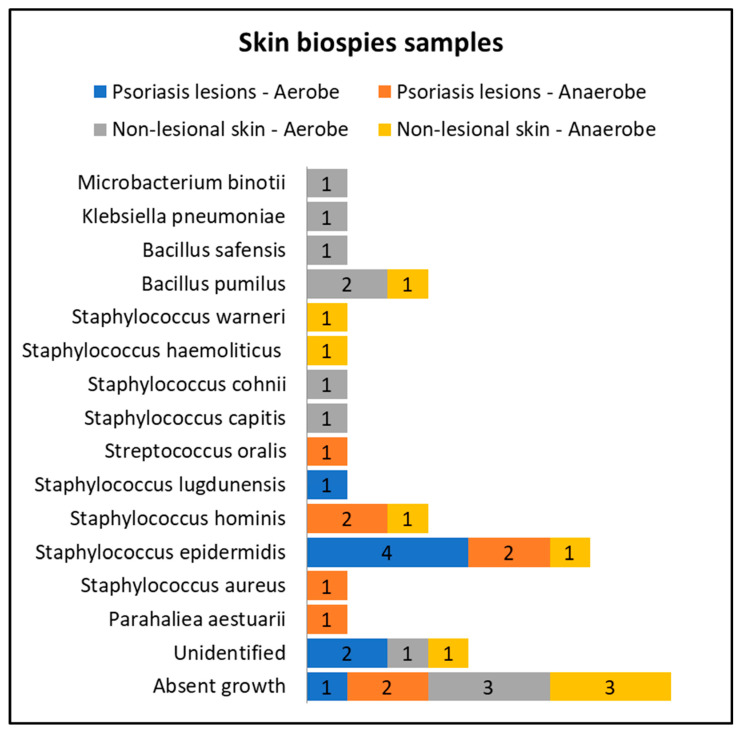
Skin biopsies sampling results from lesional skin, nonlesional sites in psoriasis patients and control groups—bacterial species isolated in aerobe and anaerobe culture mediums.

Only eight patients in the active group consented to skin biopsies, and skin samples were prelevated from both lesional (elbow and anterior trunk) and nonlesional areas (elbow and anterior trunk). The most common bacteria isolated from the skin biopsies of psoriasis lesions were *Staphylococcus epidermidis* and *Staphylococcus hominis* (Figure 3).

Cultures from skin biopsies performed from nonlesional skin of psoriasis patients revealed a higher diversity of commensal flora, each culture yielding different results (Figure 3).

Multiple bacterial strains were isolated from each sample collected from both psoriasis patients and controls. The samples collected from the psoriasis lesions presented the least diverse microbial populations. More than three different bacterial strains were isolated in 3.1% of lesional skin samples vs. 8.4% of perilesional skin samples and 13.4% of samples collected in the control group (Table 1).

The most frequent bacterial agents isolated from psoriasis lesions were *Staphylococcus epidermidis* (isolated from 36.5% of samples), *Staphylococcus aureus* (isolated from 13.5% of samples) and *Bacillus subtilis* (3.1%). The latter was only identified in samples collected from psoriasis lesions, not in nonlesional skin of psoriasis patients and controls. *Staphylococcus epidermidis* and *Staphylococcus aureus* were significantly more frequently encountered in psoriasis lesions compared with nonlesional skin of psoriasis patients and controls (Table 2).

On the other hand, *Bacillus cereus* was significantly more frequently isolated in the samples collected from the control group (15,4%) compared with the samples collected from psoriasis lesions (4.2%) and perilesional skin of psoriasis patients (3.1%) (Table 2).

The characteristics of the cutaneous microbiome, depending on the local microenvironment, were assessed. We analyzed the growth of microorganisms in skin areas with different humidity levels and observed that the more humid the microenvironment, the greater the number and diversity of the skin flora. Samples collected from humid regions, such as the axillary and inguinal folds, displayed higher numbers of microorganisms than samples collected from dry areas, both in the active and the control groups, as illustrated in Table 3 and Table 4.

**Figure 4:**
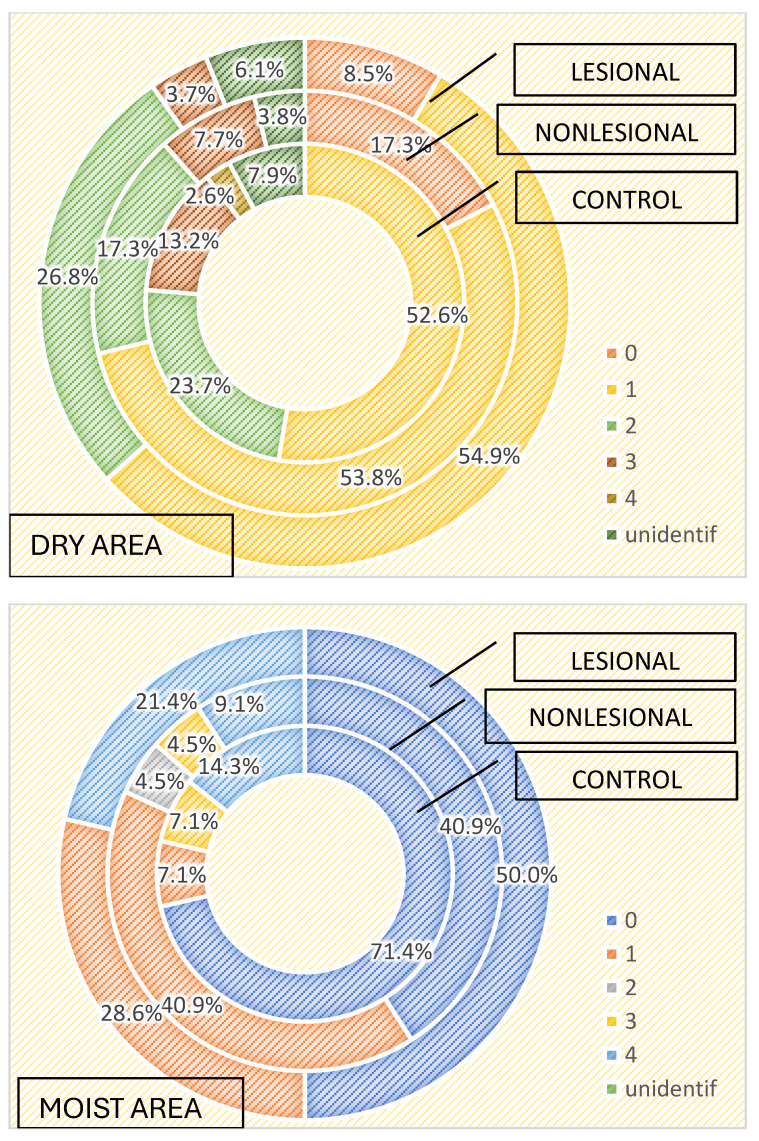
Number of strains identified in samples collected from dry and moist skin areas from psoriasis lesions, nonlesional skin of psoriasis patients and controls.

Three or more bacterial strains were isolated in 21.4% of samples collected from psoriasis lesions and 13.6% of samples collected from perilesional skin located in moist regions compared with 3.7% of samples collected from psoriasis lesions and 7.7% of samples collected from perilesional skin located in dry areas (Figure 1). The cutaneous microbiome also significantly differed in moist and dry regions in the control group, with three or more bacterial strains being isolated in 21.4% and 15.8% of samples, respectively (Figure 4).

*Staphylococcus hominis* and *Staphylococcus epidermidis* were the most frequently isolated bacteria in moist skin regions, regardless of the patient group. On the other hand, *Staphylococcus aureus* was only isolated from psoriasis lesions (28.6%) and perilesional skin (13.6%) and not in samples collected in the control group from moist regions (Table 3). *Bacillus* spp. was also significantly more common in perilesional moist skin. On the other hand, *Micrococcus luteus* and *Staphylococcus lugdunensis* were only isolated from samples collected from moist skin areas of controls. *Candida* spp. was isolated in only one sample collected from perilesional moist skin.

Regarding the qualitative results of samples collected from dry areas, statistical significance was observed for *Bacillus cereus*, which was isolated in 18.4% of samples collected from the control group, 3.8% of samples collected from the perilesional skin of psoriasis patients and 3.7% of samples from psoriasis lesions (Table 4).

In samples collected from psoriasis lesions located in dry areas, *Bacillus subtilis* and *Proteus mirabilis* were isolated. These strains were not detected in samples collected from dry areas of perilesional skin of psoriasis patients or from samples collected from the dry areas in the control group. Moreover, 39% of samples resulted in the growth of *Staphylococcus epidermidis* in lesional dry areas, statistically significant in comparison with perilesional and control samples (Table 4).

Bacteria detected in the dry perilesional sites showed higher diversity. *Bacillus pumillus* (7.7%), *Klebsiella pneumoniae* (detected only in samples from dry perilesional areas), *Staphylococcus aureus* (11.5%), *Staphylococcus hominis* and *Streptococcus* spp. were isolated in these samples.

In samples collected from dry skin areas in the control group, *Candida* spp., *Enterococcus* spp. and *Bacillus* spp. were identified.

**Figure 5:**
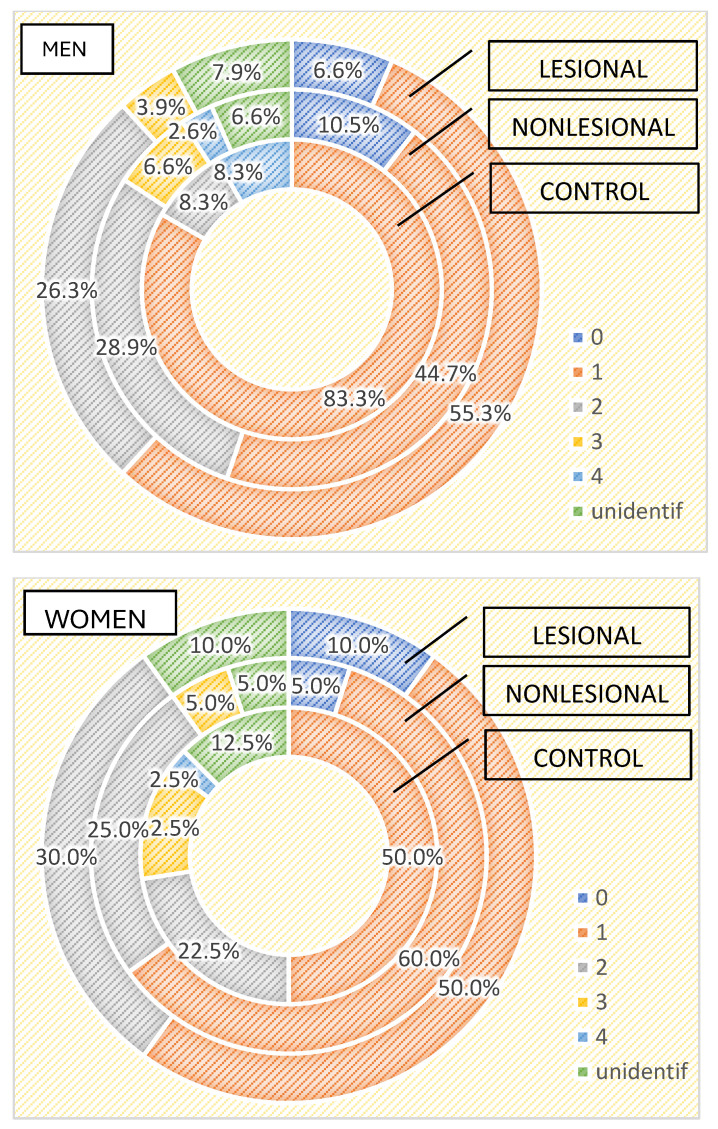
Number of strains identified in psoriasis lesions, nonlesional skin of psoriasis patients and controls in men and women.

Regarding the differences between the cutaneous microbiome in psoriasis patients and healthy individuals depending on gender, we found higher bacterial counts in male psoriasis patients in both lesional and perilesional sites (Figure 5). Contrarily, a higher number of bacteria strains were isolated in female subjects included in the control group than in men (Figure 5). Three or more bacterial strains were identified in 15% of samples collected from female individuals compared to 8.3% identified in male subjects.

The qualitative analysis showed *Staphylococcus aureus* was significantly more frequently isolated in female patients (35% developed from samples collected from psoriasis plaques and 30% from nonlesional sites of female psoriasis patients) (*p* = 0.002).

On the other hand, in male psoriasis patients included in our study, *Staphylococcus epidermidis* was significantly more frequently isolated compared to the control group (38.2% vs. 16.7% in the control group and 22.4% from nonlesional samples *p* = 0.06), whereas *Bacillus cereus* was isolated more frequently in the control group (16.7% vs. 2.6% from nonlesional samples and 3.9% from lesional areas *p* = 0.08).

**Figure 6:**
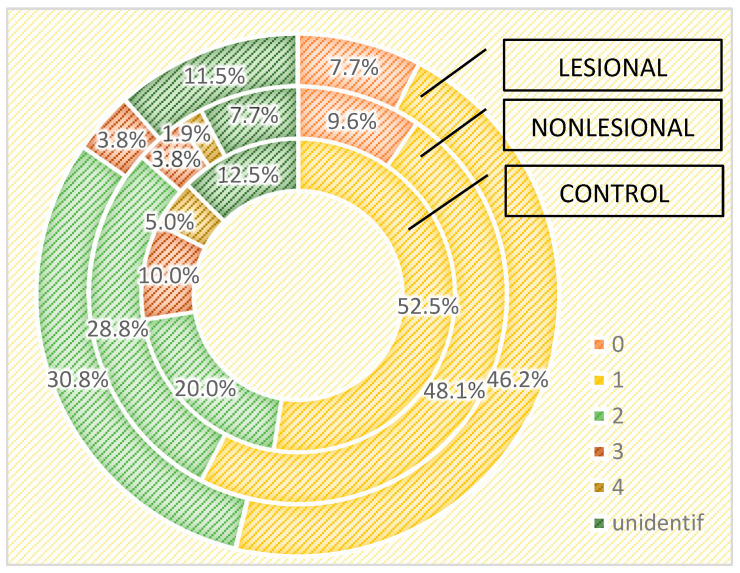
Number of strains identified in psoriasis lesions, nonlesional skin of psoriasis patients and controls living in urban areas.

**Figure 7:**
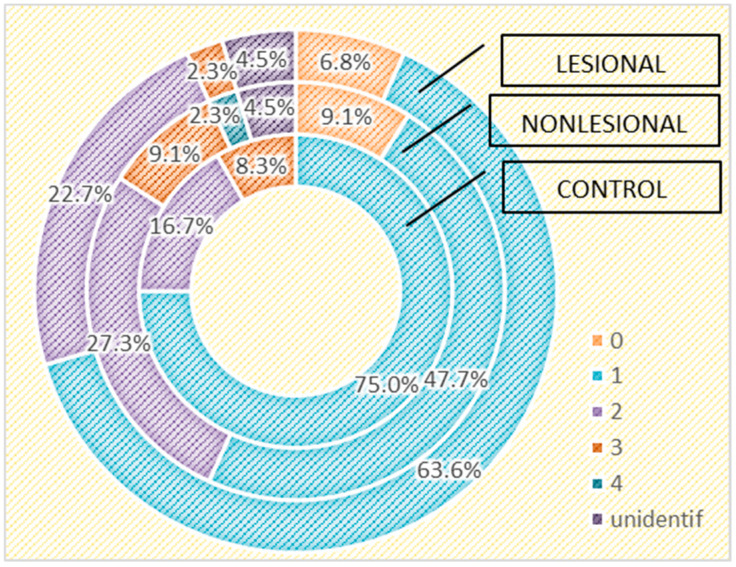
Number of strains identified in psoriasis lesions, nonlesional skin of psoriasis patients and controls living in rural areas.

We also assessed the differences in the skin microbiome in patients living in urban and rural areas. In individuals living in urban areas, we detected higher bacterial counts compared with those living in rural areas in psoriasis lesions and in the control group, but not in the perilesional skin of psoriasis patients (Figure 6 and Figure 7).

**Figure 8:**
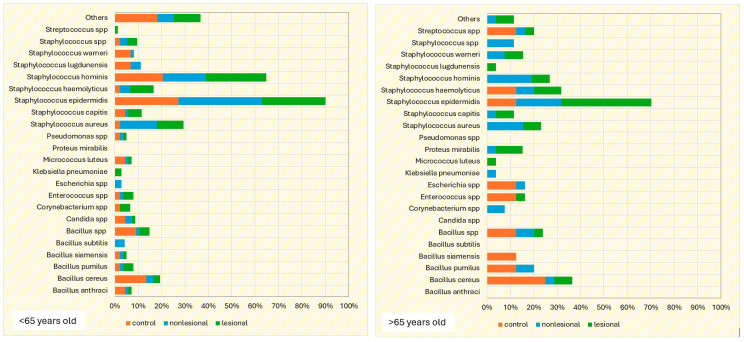
Number of bacterial strains identified in psoriasis lesions, nonlesional skin of psoriasis patients and the control group in individuals younger than 65 years and older than 65 years.

The influence of age on the cutaneous microbiome was also considered and evaluated. Psoriasis patients older than 65 presented greater quantitative changes in skin flora compared with patients younger than 65. Interestingly, analysis of the control group yielded opposite results: 15.4% of the samples collected from perilesional sites developed three or more bacterial strains in patients over 65 years old, significantly higher than younger subjects (5.7%) (Figure 8).

Further, we assessed the changes in the cutaneous microbiome depending on the severity of psoriasis. Higher PASI and NAPSI scores were registered in male than in female participants, and both scores were higher in patients living in rural areas. PASI scores ranged between 2 and 43.9, with a mean of 18.940 ± 12.9729 standard deviation. In male patients, the mean PASI score was 20.407 ± 12.7331 standard deviation compared to 14.540 ± 14.1307 standard deviation in female patients. In rural areas, the mean values of PASI were 23.011 ± 13.9839 standard deviation, significantly higher than the mean PASI score of 15.609 ± 11.6706 standard deviation of patients living in urban areas (Table 5).

NAPSI values ranged between 0 and 40 units, with a mean of 9.45 ± 12.412 standard deviation. Higher values were scored by men (mean NAPSI 9.80 ± 13.702 standard deviation) than female patients (mean NAPSI 12.11 ± 16.707 standard deviation) and by patients living in rural areas (mean NAPSI 12.11 ± 16.707 standard deviation) compared with patients from urban areas (mean NAPSI 7.27 ± 7.604 standard deviation) (Table 6).

ESIF and PSSI scores were higher in female psoriasis patients living in urban areas, while DLQI scores were higher in female psoriasis patients from rural areas. ESIF mean values for female patients were 8.00 ± 12.329 standard deviation compared to 7.20 ± 8.283 standard deviation in men, and 8.09 ± 10.064 standard deviation for patients from urban areas compared with 6.56 ± 8.263 standard deviation for patients from rural regions. PSSI values ranged between 0 and 54. In female patients, the mean PSSI value was 17.00 ± 20.952 standard deviation, significantly higher compared to men, whose mean PSSI value was 8.87 ± 9.920 standard deviation. The mean PSSI of patients from urban areas was 14.36 ± 15.977 standard deviation, more than twice greater than that of patients living in rural areas (6.67 ± 8.185 standard deviation).

Regarding DLQI scores, values ranged between 2 and 28. In women patients, the mean values were 19.40 ± 6.914 standard deviation, higher than in men (14.20 ± 7.664 standard deviation). In patients from rural areas, the mean DLQI value was 17.22 ± 7.242 standard deviation, higher than in patients from urban areas (14.09 ± 8.043 standard deviation) (Table 7).

**Figure 9:**
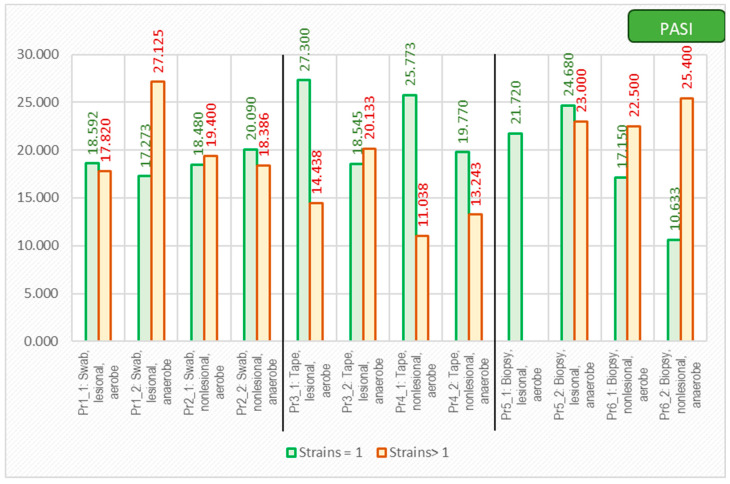
The correlation between PASI score and number of isolated strains using different sampling techniques.

As expected, the PASI score correlated with a lower diversity of the cutaneous microbiome, with only one bacterial strain being isolated from the lesions of the most severe psoriasis patients (Figure 9).

## 3. Discussion

Advanced technology such as next-generation sequencing, shotgun metagenomics, metatranscriptomics, metaproteomics and metabolomic approaches have enabled the study of the structure and function of human microbiomes, opening a myriad of research avenues and leading to an impressive advancement of our knowledge regarding the role of the microbiome in both health and disease states [16,18].

Keratinocytes express aryl hydrocarbon receptors, which are activated by bacteria found on human skin. This receptor has an important role in maintaining the cutaneous barriers’ integrity and epithelial recovery after trauma [19]. Commensal bacteria play a pivotal role in maintaining the barrier function of the cutaneous tissue by producing enzymes such as sphingomyelinase, secreted by *Staphylococcus epidermidis*. Sphingomyelinase induces the secretion of ceramides in the stratum corneum, reducing transepidermal water loss and improving the lipid layer of the skin [20].

In a sebum-rich microenvironment such as the follicular unit, bacteria such as *Cutibacterium* spp. and *Corynebacterium* spp. secrete lipases that dissolve sebum triglycerides. The ensuing free fatty acids lower the skin’s pH, an efficient defense mechanism against pathogenic infectious agents [21,22]. Commensal bacteria act as positive competitors for pathogenic microorganisms, resulting in increased production of antimicrobial peptides and biofilm synthesis [23].

Another role of the commensal flora resides in the modulation of the innate immune response through the release of proinflammatory cytokines such as IL-1α, as well as C5a receptor for complement and LL-37, β defensins and perforin-2. These molecules are able to bind to Toll-like receptors [23,24]. Proliferation of cutaneous γδ T cells, which produce IL-17 and provide protection against *Candida albicans* and *Staphylococcus epidermidis*, is induced by *Corynebacterium accolens* [25]. Since γδ T cells have been shown to secrete IL-17A without IL-23 activation, this emphasizes the significance of the skin microbiome in the initiation or exacerbation of psoriasis [26]. The adaptive immune response is also significantly influenced by the skin microbiome. Commensal bacteria stimulate cytotoxic (TC17) and helper (Th17) T cells to produce IL-17A. These cells develop into memory cells that reside in the skin and aid in tissue healing and protection against pathogens, but they are also responsible for the recurrence of psoriasis lesions upon exposure to eliciting factors [27].

Recent studies highlight important differences between the skin microbiome of healthy individuals and psoriasis patients [28,29,30,31]. Our study provides new evidence that supports this theory, as the results point to major qualitative and quantitative changes in the cutaneous microbiome of psoriasis patients compared with healthy controls. In concordance with the study of the skin microbiome in psoriasis by Alekseyenko et al., we also observed lower diversity and fewer bacterial strains from psoriatic plaque samples [30]. Our results highlight that samples collected from psoriatic plaques, using all three methods, generated lower diversity and microbial agents than samples collected from nonlesional sites or from control individuals. These results are in contrast to other studies that reflect that the skin microbiome of psoriasis patients differs from that of healthy subjects, not only in lesional but also in nonlesional skin, showing greater heterogeneity [28,29,30]. Some studies concluded that the skin microbiome of psoriasis patients is characterized by the predominance of *Streptococcus* spp. and *Firmicutes* spp. [28,30,32,33], increased concentrations of *Coprobacillus* spp., *Ruminococcus* spp. [32,33], *Corynabacterium* spp. [28,30,34,35] and *Proteobacteria* spp. [28,36] and fewer *Actinobacteria* spp., *Bacteroides* spp., and *Cutibacterium* spp. compared with healthy skin [28,32,33,37]. Increased density of *Staphylococcus* spp. in both psoriasis lesions and apparently normal skin was reported by most studies [29,30,38,39,40], but not all studies [28]. In our study, a higher count of *Bacillus* spp., *Staphylococcus aureus* and *Staphylococcus epidermidis* was found in psoriasis lesions compared with nonlesional sites of psoriasis patients and controls. *Bacillus pumillus*, *Corynebacterium* spp., *Klebsiella pneumoniae* and *Staphylococcus* spp. were found in greater numbers in samples collected from the perilesional skin of psoriasis patients, whereas in the control group, Bacillus spp., *Enterococcus* spp. and *Micrococcus luteus* were more frequently isolated than in psoriasis patients.

Regarding fungal colonization, we detected higher *Candida* spp. counts in samples collected from nonlesional skin sites of psoriasis patients located in moist regions in male psoriasis patients compared with controls. Our results are in accordance with previous studies that showed *Candida albicans* is present in large numbers in psoriasis lesions, especially in patients with inverse psoriasis [41], and may be involved in the persistence and exacerbation of the disease by stimulating Candida-sensitized αβ T cells to express IL-17 [42,43,44,45]. In addition, β-glucan expressed by *Candida* spp. is recognized by PRRs on mDCs, stimulating the production of IL-36α, a key cytokine in the pathogenesis of pustular psoriasis [46]. Antifungals improve the aspect of psoriasis-affected nails, which supports the implication of fungi in the exacerbation of psoriasis lesions [47].

The composition and function of the skin microbiome considerably varies in different anatomic sites, as our results suggest, depending on the lipid layer, skin pH, the particularities of perspiration and sebum production, humidity, local skin temperature, and the region’s exposure to light [46]. The samples we collected from moist areas such as the axillary, inguinal, and submammary folds were colonized by higher numbers of bacteria and fungi than samples collected from dry areas in both psoriasis patients and controls. In addition, samples collected from humid skin areas of psoriasis patients revealed higher colonization of *Corynebacterium* spp., *Pseudomonas* spp., *Enterococcus* spp. and *Candida* spp. than samples collected from dry areas. In the control group, the samples collected from moist areas revealed higher numbers of *Staphylococus* spp. and *Micrococcus* spp., whereas in dry areas, we detected higher numbers of *Candida* spp., *Bacillus* spp. and *Enterococcus* spp. Our results are similar to the results of other studies in which moist intertriginous areas are preferentially colonized by *Corynebacterium* spp., *β-Proteobacteria* spp. and *Staphylococcus* spp. and dry areas by *Corynebacterium* spp., *β-proteobacteria* spp. and *Flavobacterium* spp. [48]. As demonstrated by previous research, the microbiome of areas rich in sebaceous glands is characterized by less diversity and higher densities of *Cutibacterium* spp. and *Staphylococcus* spp. [49]. A series of fungal species also populate normal skin, the most common of which is *Malassezia* spp., especially on the cephalic extremity, trunk and upper limbs [50]. Due to the favorable local conditions, a larger variety of fungi colonize the skin of the feet, among them *Malassezia* spp., *Aspergillus* spp., *Cryptococcus* spp., *Rhodotorula* spp. and *Epicoccum* spp. [50]. *Demodex* spp., a microscopic mite, is also part of the commensal flora of pilosebaceous follicles and is mainly found on the face [51,52]. The microbiome of the skin surface significantly differs from that of the pilosebaceous unit. The latter is poorly characterized mainly due to frequent sample contamination [53]. The role of the microbiome in the homeostasis of the hair follicle is incompletely understood, but a series of recent studies have offered valuable new data on the modulatory effects of commensal flora on hair follicle functions [53,54,55,56]

The pilosebaceous unit is particularly rich in *Cutibacterium acnes* and *Malassezia restricta* but also harbors high numbers of *Staphylococcus epidermidis*, archaea and viruses [53].

The microbiome modulates the hair follicle’s physiology, from its development to its complex metabolic functions [53]. Considering the important influence of commensal flora on tissue regeneration, it is noteworthy that following skin injuries, tumor necrosis factor (TNF)-α induces AKT/β-catenin signaling in the stem cells of hair follicles, which promotes normal hair follicle development and neogenesis [54].

TNF-α is released by macrophages recruited to the wounded site by transforming growth factor (TGF)-β1 and CX3CR1 [55]. Moreover, as demonstrated by Chen et al., Akt/NF-κB signaling also mediates skin aging, associated with a higher risk of cell transformation [56].

Differences between male and female cutaneous microbiomes have been suggested by different studies [57,58]. Considering the variations in the number and activity of sweat glands due to different hormonal profiles and hygienic routines, male and female cutaneous microbiomes may prove distinct. Our study revealed important gender differences in the skin microbiome. In the control group, we isolated higher numbers of microorganisms in samples collected from women than men. On the contrary, we detected a higher bacterial colonization in male psoriasis patients compared to female patients with psoriasis. These results might be explained by the different daily skincare cosmetic routines of female psoriasis patients, who tend to be more rigorous in applying local treatments due to cosmetic reasons [59,60]. Robert et al. reported that facial microbial taxa richness was lower in men than women, given the particular facial microenvironment of male individuals [57].

The skin microbiome varies with age, changing dramatically around puberty, given the stimulating effect of androgens on sebaceous glands, which favors the proliferation of lipophilic bacteria, mainly *Cutibacterium* spp. and *Malassezia* spp. [48,49,50,61]. During adulthood, the skin microbiome is relatively stable [49] unless influenced by environmental exposures, including diet, hygiene, antimicrobial treatments, comorbidities, use of cosmetics or topical drugs, climate and light exposure [48,49,62,63,64]

The alterations of the skin in older individuals due to the decrease in collagen synthesis and reduced sebum production lead to changes in the skin microbiome. Multiple studies have demonstrated that the diversity of the cutaneous microbiome increases with age [65,66,67,68]. A greater abundance of *Streptococcus* spp., *Corynebacterium* spp. and *Acinetobacter* spp. has been related to older age [66,69]. Contrarily, due to decreased sebum production, a decrease in *Cutibacterium* spp. colonies in the elderly has been detected [66,69]. In our study, we found fewer microorganisms in the skin samples collected from controls older than 65 years. Our results are in contrast to recent studies that highlight the fact that with age, given the modifications in sweat and sebum levels, the diversity of the cutaneous microbiome increases. Nevertheless, we observed higher microorganism diversity and quantitative changes in the bacterial cultures of samples collected from psoriasis lesions and nonlesional skin of psoriasis patients in patients over 65 years compared with younger patients, especially in samples collected from perilesional skin. In patients older than 65, we isolated higher amounts of *Bacillus* spp., *Corynebacterium* spp, *Staphylococcus* spp. and *Micrococcus* spp.

The composition of the cutaneous microbiome is greatly influenced by the living environment. External factors such as resulting in human-specific microorganisms mainly colonizing the skin. Living in temperature and cultural and working habits impact the skin microbiome. Living in urban environments modulates the cutaneous microbiome, the predominant human–human interactions rural areas enriches the cutaneous microbiome with agents found in soil and animals [70,71]. In our study, higher bacterial diversity was observed in skin samples collected from people living in urban areas than in rural environments. Given the characteristics of the living environment of the study population, the region being mostly industrial, even in rural areas, the cutaneous microbiome mostly reflects human–human interactions.

Few studies have assessed the cutaneous microbiome and severity scores. In the study of Buhaș et al., lower severity scores were observed when applying topical therapy together with pre- and probiotics in the management of psoriasis [72]. Upon analyzing psoriasis severity scores, we observed that higher PASI and NAPSI scores were reported by male participants than female patients and that both scores were higher in individuals living in rural areas. ESIF, DLQI and PSSI were higher in female participants and individuals living in urban areas, except for DLQI, which was higher in rural living individuals.

## 4. Materials and Methods

We performed a retrospective case-control study, which included a total of 33 individuals older than 18 who were referred to our clinic between May and July 2023. Each participant in the study signed a written informed consent. The study methodology was approved by the Ethical Committees of our hospital and university.

**Figure 10:**
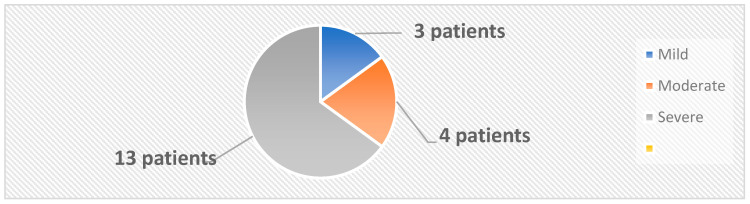
Classification of patients based on PASI score.

The 33 subjects were classified into two categories: the active group, comprising 20 patients diagnosed with psoriasis, and the control group, which included 13 healthy individuals. The mean age and the female/male ratio were similar in the active and control groups. A diagnosis of psoriasis was established based on clinical examination and confirmed by histopathological examination. Swab skin samples were collected from both the active and the control groups. In the active group, 4 samples were collected from each patient, 2 from psoriatic plaques and 2 from the nonlesional skin. In the control group, 2 samples per patient were collected from body regions similar to those from the active group. The samples were inoculated on both aerobe and anaerobe mediums. The severity of psoriasis was evaluated using 5 validated psoriasis severity scores, i.e., Psoriasis Area and Severity Index (PASI), Nail Psoriasis Severity Index (NPSI), Erythema Scaling Induration Fissuring Scale (ESIFS), Psoriasis Scalp Severity Index (PSSI) and Dermatology Life Quality Index (DLQI). Patients were classified as having mild, moderate or severe psoriasis, as depicted in Figure 10.

The exclusion criteria for the control group were represented by a personal history of neoplastic and/or inflammatory skin diseases and a family history of psoriasis. Patients and controls using local or systemic antibiotic therapy, immunomodulators or prebiotics for 3 months prior to sample collection were also excluded from the study.

The study of the cutaneous microbiome was performed using several skin sampling techniques from both lesional and perilesional sites. The patients included in the study were advised to use only mild soap and emollients two weeks before the sampling process and were asked not to shower 24 h prior to sample collection. In the active group, we performed three types of skin samplings: tape stripping, collection of swab samples and cutaneous punch biopsies. In the healthy control group, we collected swab and tape-stripping skin samples.

Tape-stripping samples were collected using 2 × 4 cm sterile adhesive tape, which was applied directly to the lesional and nonlesional skin sites. The skin biopsies were performed under local anesthesia using 1% lidocaine, without the prior application of antiseptic solutions, using a 3 mm biopsy punch. After collection, the samples were inoculated on both aerobe and anaerobe mediums.

A total of 12 skin samplings were performed on each psoriasis patient, as follows: 4 swab samples (2 from lesional sites and 2 from nonlesional sites), 4 adhesive tape samples (2 from lesional sites and 2 from nonlesional sites) and 4 skin biopsies (2 from lesional sites and 2 from nonlesional sites), inoculated on both aerobe and anaerobe mediums. Only 8 patients consented to skin punch biopsies for the study of the cutaneous microbiome. In the control group, a total of 4 skin samples were collected from each individual (2 swab samples, inoculated in aerobe and anaerobe medium, respectively, and 2 tape-stripping skin samples, inoculated in aerobe and anaerobe medium, respectively). The samples were collected from body regions similar to those collected in the active group.

Glucose nutrient broth medium (Thermo Fischer Scientific, Basingstoke, Oxoid Ltd., Hampshire, UK) was used for the aerobic cultures and fluid thioglycolate medium (Thermo Fischer Scientific, Basingstoke, Oxoid Ltd. Hampshire, UK) for the anaerobic ones. Further, the bacterial colonies were cultured onto agar with 5% sheep blood (Thermo Fischer Scientific, Basingstoke, Oxoid Ltd., Hampshire, UK) and incubated for 24 h in aerobic and anaerobic conditions at 37 °C. For the identification of bacterial isolates, MALDI-TOF with Auto MS 100-Mass Spectrometer (Autobio Diagnostics Co., Ltd., Zhengzhou, China) was employed. Quality control was performed using the following reference strains in the isolation and identification process: ATCC 19615, *Streptococcus pyogenes*; ATCC 25923, *Staphylococcus aureus*; ATCC 25922, *Escherichia coli*; ATCC 27853, *Pseudomonas aeruginosa*; and ATCC 10231, *Candida albicans* (Thermo Fisher Scientific, Lenexa, KS, USA).

The statistical analysis was performed using SPSS 29.0. The qualitative data are presented as absolute frequencies and percentages, and the quantitative data are presented as averages and standard deviations. Chi-squared and Fisher tests were used to reveal the associations between qualitative data. *p*-values of <0.05 were considered statistically significant, while *p*-values of <0.01 were regarded as highly statistically significant.

## 5. Conclusions

Our study contributes to the growing evidence regarding important changes in the skin microbiome of psoriasis patients. The implications of skin dysbiosis in psoriasis pathogenesis continue to attract researchers’ attention, and the underlying mechanisms are starting to unfold. Modulation of the skin microbiome represents an interesting adjuvant strategy in the personalized management of psoriasis. Larger standardized studies are needed in order to elucidate the microbiome changes in psoriasis patients, clarify their role in the pathogenesis of psoriasis, decipher the interactions between the commensal microorganisms of the same and different niches and between microbiomes and the host and identify new therapeutic strategies.

## Figures and Tables

**Table 1:** Number of bacterial strains identified from the samples collected from the control group, psoriasis lesions and nonlesional skin of psoriasis patients.

			Control		Lesional		Nonlesional		Pearson Chi2 Test
			Number of Strains	%	Number of Strains	%	Number of Strains	%	
TOTAL	Number of strains	0			7	7.3%	9	9.4%	Chi2 = 12.819
		1	30	57.7%	52	54.2%	46	47.9%	*p* = 0.234
		2	10	19.2%	26	27.1%	27	28.1%	
		3	5	9.6%	3	3.1%	6	6.3%	
		4	2	3.8%			2	2.1%	
		Unidentified	5	9.6%	8	8.3%	6	6.3%	
	Total		52	100.0%	96	100.0%	96	100.0%	

**Table 2:** The most frequent bacterial agents isolated from psoriasis lesions, nonlesional skin of psoriasis patients and controls.

	Psoriasis Lesions	Nonlesional Skin of Psoriasis Patients	Controls
*Bacillus subtilis*	3 (3.1%)	0	0
*Staphylococcus aureus*	13 (13.5%)	12 (12.5%)	1 (1.9%)
*Staphylococcus epidermidis*	35 (36.5%)	24 (25%)	13 (25%)
*Bacillus cereus*	4 (4.2%)	3 (3.1%)	8 (15.4%)
*Klebsiela pneumoniae*	0	3 (3.1%)	0

**Table 3:** Comparative analysis of the bacterial strains identified in samples collected from moist skin areas from psoriasis lesions, nonlesional skin of psoriasis patients and controls.

		Control		Lesional		Nonlesional		Pearson Chi 2 Test
		Number of Strains	%	Number of Strains	%	Number of Strains	%	
MOIST REGION	*Bacillus anthraci*							-
	*Bacillus cereus*	1	7.1%	1	7.1%	1	2.3%	*p* = 0.602
	*Bacillus pumilus*					1	2.3%	*p* = 0.724
	*Bacillus siamensis*					1	2.3%	*p* = 0.724
	*Bacillus subtilis*							-
	*Bacillus* spp.					2	4.5%	*p* = 0.520
	*Candida* spp.					1	2.3%	*p* = 0.724
	*Corynebacterium* spp.	1	7.1%			5	11.4%	*p* = 0.401
	*Enterococcus* spp.			1	7.1%	2	4.5%	*p* = 0.627
	*Escherichia* spp.							-
	*Klebsiella pneumoniae*					2	4.5%	*p* = 0.520
	*Micrococcus luteus*	1	7.1%					*p* = 0.122
	*Proteus mirabilis*					1	2.3%	*p* = 0.724
	*Pseudomonas* spp.					1	2.3%	*p* = 0.724
	*Staphylococcus aureus*			4	28.6%	6	13.6%	*p* = 0.091
	*Staphylococcus capitis*					2	4.5%	*p* = 0.520
	*Staphylococcus epidermidis*	2	14.3%	3	21.4%	13	29.5%	*p* = 0.487
	*Staphylococcus haemolyticus*	1	7.1%			5	11.4%	*p* = 0.401
	*Staphylococcus hominis*	4	28.6%	4	28.6%	14	31.8%	*p* = 0.958
	*Staphylococcus lugdunensis*	2	14.3%					*p* = 0.014
	*Staphylococcus warneri*	1	7.1%			1	2.3%	*p* = 0.489
	*Staphylococcus* spp.					5	11.4%	*p* = 0.181
	*Streptococcus* spp.							-
	Others	3	21.4%	2	14.3%	3	6.8%	*p* = 0.290
	Total	14	100.0%	14	100.0%	44	100.0%	

**Table 4:** Comparative analysis of the bacterial strains identified in samples collected from dry skin areas from psoriasis lesions, nonlesional skin of psoriasis patients and controls.

		Control		Lesional		Nonlesional		Pearson Chi2 Test
		Number of Strains	%	Number of Strains	%	Number of Strains	%	
DRY REGION	*Bacillus anthraci*	2	5.3%	1	1.2%	1	1.9%	*p* = 0.382
	*Bacillus cereus*	7	18.4%	3	3.7%	2	3.8%	*p* = 0.007
	*Bacillus pumilus*	2	5.3%	1	1.2%	4	7.7%	*p* = 0.166
	*Bacillus siamensis*	2	5.3%	1	1.2%			*p* = 0.150
	*Bacillus subtilis*			3	3.7%			*p* = 0.187
	*Bacillus* spp.	5	13.2%	2	2.4%	3	5.8%	*p* = 0.066
	*Candida* spp.	2	5.3%	2	2.4%			*p* = 0.261
	*Corynebacterium* spp.							-
	*Enterococcus* spp.	2	5.3%	1	1.2%	1	1.9%	*p* = 0.382
	*Escherichia* spp.	1	2.6%	2	2.4%	1	1.9%	*p* = 0.972
	*Klebsiella pneumoniae*					1	1.9%	*p* = 0.313
	*Micrococcus luteus*	1	2.6%	2	2.4%	1	1.9%	*p* = 0.972
	*Proteus mirabilis*			3	3.7%			*p* = 0.187
	*Pseudomonas* spp.	1	2.6%	1	1.2%			*p* = 0.515
	*Staphylococcus aureus*	1	2.6%	9	11.0%	6	11.5%	*p* = 0.275
	*Staphylococcus capitis*	2	5.3%	3	3.7%	3	5.8%	*p* = 0.835
	*Staphylococcus epidermidis*	11	28.9%	32	39.0%	11	21.2%	*p* = 0.088
	*Staphylococcus haemolyticus*	1	2.6%	6	7.3%	4	7.7%	*p* = 0.559
	*Staphylococcus hominis*	5	13.2%	11	13.4%	9	17.3%	*p* = 0.793
	*Staphylococcus lugdunensis*	1	2.6%	4	4.9%			*p* = 0.260
	*Staphylococcus warneri*	2	5.3%	3	3.7%	1	1.9%	*p* = 0.690
	*Staphylococcus* spp. *(altele)*	1	2.6%	2	2.4%	1	1.9%	*p* = 0.972
	*Streptococcus* spp.	1	2.6%	1	1.2%	2	3.8%	*p* = 0.611
	Others	5	13.2%	5	6.1%	6	11.5%	*p* = 0.372
	Total	38	100.0%	82	100.0%	52	100.0%	

**Table 5:** PASI score analysis—descriptive statistics and comparative study.

		Mean	Std. Error of Mean	Std. Deviation	Min	Max	Median	t-Student/Mann–Whitney Test
PASI	Total	18.940	2.9008	12.9729	2.0	43.9	14.700	
	Male	20.407	3.2877	12.7331	4.1	43.9	15.000	t = 0.870
	Female	14.540	6.3195	14.1307	2.0	35.1	10.000	*p* = 0.396
	Urban	15.609	3.5188	11.6706	2.0	35.1	14.400	t = −1.292
	Rural	23.011	4.6613	13.9839	7.0	43.9	25.400	*p* = 0.213

**Table 6:** NAPSI score analysis—descriptive statistics and comparative study.

		Mean	Std. Error of Mean	Std. Deviation	Min	Max	Median	t-Student/Mann–Whitney test
NAPSI	Total	9.45	2.775	12.412	0	40	4.00	
	Male	9.80	3.538	13.702	0	40	0.00	U = 35.00
	Female	8.40	3.816	8.532	0	20	10.00	*p* = 0.852
	Urban	7.27	2.293	7.604	0	20	8.00	U = 47.000
	Rural	12.11	5.569	16.707	0	40	0.00	*p* = 0.839

**Table 7:** DLQI score analysis—descriptive statistics and comparative study.

		Mean	Std. Error of Mean	Std. Deviation	Min	Max	Median	t-Student/Mann–Whitney test
DLQI	Total	15.50	1.713	7.661	2	28	15.00	
	Male	14.20	1.979	7.664	2	28	15.00	t = −1.342
	Female	19.40	3.092	6.914	10	25	24.00	*p* = 0.196
	Urban	14.09	2.425	8.043	2	25	14.00	t = −0.905
	Rural	17.22	2.414	7.242	5	28	15.00	*p* = 0.377

## Data Availability

Data is contained within the article.
